# Utilization of Anti-Seizure Medication (ASMS) and Seizure Control in Pediatric Epilepsy: A Retrospective Cross-Sectional Study in Oman

**DOI:** 10.3390/healthcare14182910

**Published:** 2026-09-08

**Authors:** Yousra Nomier, Yaqeen Al-Aamri, Rawan AL-Toqi, Abdallah Al-Nofli, Ibrahim Al-Sibayi, Yahya Al-Farsi, Ibrahim Al-Zakwani

**Affiliations:** 1 Pharmacology and Clinical Pharmacy Department, College of Medicine and Health Science, Sultan Qaboos University, P.O. Box 35, Muscat 123, Oman; azakwani@squ.edu.om; 2 College of Medicine and Health Science, Sultan Qaboos University, P.O. Box 35, Muscat 123, Oman; 3 Epidemiology & Public Health Department, College of Medicine and Health Science, Sultan Qaboos University, P.O. Box 35, Muscat 123, Oman; ymfarsi@squ.edu.om

**Keywords:** pediatric epilepsy, anti-seizure medications, medication adherence, seizure control, adverse effects, drug utilization, Oman

## Abstract

**Objectives**: Epilepsy is a chronic neurological disorder associated with substantial morbidity, reduced quality of life, and a considerable global healthcare burden. We aimed to assess anti-seizure medication (ASMS) utilization patterns, adherence levels, and factors influencing seizure control and adverse effects among pediatric patients with epilepsy in Oman. **Methods**: A retrospective cross-sectional study was conducted at Sultan Qaboos University Hospital (SQUH) between January 2023 and November 2024. A total of 305 pediatric patients aged 2–12 years receiving at least one ASMS with a minimum of two years of follow-up were included. Data were extracted from electronic medical records. Adherence was assessed using the Medication Possession Ratio (MPR). Statistical analysis was performed using SPSS. **Results**: Levetiracetam (68.52%) was the most prescribed ASMS, followed by sodium valproate (22.62%) and topiramate (8.52%). Adherence was observed in 54.68% of patients. Mean seizure duration decreased progressively across follow-up visits. Seizure control was achieved in 43% of patients. Patients without adverse effects had significantly better seizure control (56.2% vs. 10.2%, *p* < 0.001). Dose increases were associated with higher rates of adverse effects. **Conclusions**: Levetiracetam was the most commonly prescribed ASMS. Seizure type influenced drug selection. Adverse effects had a strong negative impact on seizure control, while adherence showed a limited association, indicating that managing side effects is crucial for effective treatment. Optimizing ASMS dosing and minimizing adverse effects are essential to improving outcomes in pediatric epilepsy. Dose adjustments, particularly dose increases, were associated with a higher frequency of adverse effects. Careful dose optimization may therefore be important to balance tolerability and seizure management. In this study, just over half of the patients evaluated (54.68%) were classified as adherent based on Medication Possession Ratio.

## 1. Introduction

Epilepsy is a long-term neurological disorder that affects about 50 million people around the world. It is more common in low- and middle-income countries [1]. Epilepsy is defined by the International League Against Epilepsy (ILAE) as any of the following: at least two unprovoked (or reflex) seizures occurring more than 24 h apart; one unprovoked (or reflex) seizure with a probability of further seizures similar to the general recurrence risk over the next 10 years; or the diagnosis of epilepsy syndrome [2]. A seizure is defined as a transient occurrence of signs and/or symptoms due to abnormal, excessive or synchronous neuronal activity in the brain [2]. The primary aim of anti-seizure medication (ASMS) is to reduce seizure frequency [2], as these seizures may significantly impact the daily lives of patients, affecting their physical, cognitive, and emotional well-being [3]. Children with epilepsy experience remarkably higher rates of behavioral, social, emotional, and academic difficulties compared to youth with other chronic conditions [4]. Recurrent seizures can lead to a wide range of clinical manifestations, which can profoundly impact patients’ daily lives [3].

A study in Germany found that 68% of children and adolescents with epilepsy adhered to their ASMSs [5]. A study at Riyadh National Hospital in Saudi Arabia from December 2011 to January 2014 also looked at how well adolescents aged 13 to 18 followed their ASMSs. The research identified several critical factors influencing patient adherence, including the number of medications prescribed, maternal age, the stability of parental marriage, familial support, the consistency of the relationship between healthcare providers and parents, and the frequency of seizures. The primary reasons for non-adherence are forgetfulness and apprehension over undesirable effects [6].

The selection of ASMSs is crucial to improving symptoms and preventing seizures. Factors such as patient demographics (age, sex, and pregnancy status), type of epilepsy, comorbidities, acute and chronic adverse effects, and effectiveness of the therapeutic agent, appropriate target dose, and gradual dose titration should be considered [2]. For example, sodium valproate is avoided in pregnancy due to teratogenicity risks [7]. Furthermore, carbamazepine could exacerbate certain psychiatric conditions, and potential drug–drug interactions must be considered when selecting an appropriate medication [8], especially in patients with a history of mood disorders or those taking other psychotropic medications. In 80% of patients with epilepsy, the seizures are well controlled by ASMS treatment. However, chronic drug-resistant epilepsy represents 20–30% of patients and thus prevents optimal control of these seizures [9].

The effectiveness of ASMSs in controlling seizures and the prevalence of adverse effects among patients with epilepsy have been a global concern due to the high burden of epilepsy and its impact on quality of life. The effectiveness of anti-seizure medications is strongly influenced by adherence, among other clinical factors. The magnitude of this problem is underscored by a study conducted in Oman at SQUH involving 203 confirmed patients with epilepsy, in which data were reviewed in 2003. The study reveals that while 61% of patients achieved seizure control with ASMS monotherapy, only 34% maintained this control throughout a median of a six-year follow-up period. Additionally, treatment retention rates varied among different ASMSs, with sodium valproate (VPA) demonstrating the highest retention rate (91%) over the last two years, followed by carbamazepine (CBZ) and phenytoin (PHT). However, in 67% of patients, adverse drug reactions were reported, predominantly associated with VPA [10,11]. Milligan’s study emphasized that ASMSs such as valproate, carbamazepine, and phenobarbital can cause fatigue, dizziness, and behavioral changes [12]. Al Rajeh et al.’s research found that discontinuation rates in Arab populations were high due to drug intolerance [3]. Lehmann et al. (2014) reported that adverse effects are an important barrier to long-term medication adherence, particularly in younger patients [13]. This study was informed by a pharmacoepidemiologic framework in which anti-seizure medication selection, adherence, adverse effects, and seizure control are viewed as interrelated components of treatment effectiveness in pediatric epilepsy. Although this study was conducted in Oman, the clinical challenges surrounding epilepsy management, anti-seizure medication utilization, and treatment optimization are shared across many healthcare systems worldwide. Therefore, evaluating these patterns in the Omani setting may provide insights that are relevant to broader international efforts to improve epilepsy care.

## 2. Methodology

### 2.1. Study Design and Setting

This retrospective cross-sectional study was conducted at the Neurology Department of Sultan Qaboos University Hospital (SQUH), a tertiary care center in Muscat, Sultanate of Oman. Data were collected between January 2023 and November 2024. This retrospective study was conducted using electronic medical records of pediatric patients with epilepsy followed at Sultan Qaboos University Hospital (SQUH). The study included repeated assessments across multiple follow-up visits over a two-year period.”

### 2.2. Study Hypotheses

This study was designed to test the following hypotheses:

1. There is a significant association between anti-seizure medication (ASMS) utilization patterns and seizure control outcomes in pediatric patients.
2. Medication adherence is positively associated with improved seizure control and longer seizure-free duration.
3. The presence of ASMS-related adverse effects is associated with poorer seizure control and reduced adherence.
4. Dose adjustments of ASMSs during follow-up are significantly associated with both seizure control and the occurrence of adverse effects.

### 2.3. Study Population

The study included 305 pediatric patients aged 2–12 who were diagnosed with epilepsy and received at least one ASMS with a minimum of 2 years of follow-up. Patients were excluded if they were lost to follow-up, received part of their treatment outside Oman, or had epilepsy that was managed with nonpharmacological methods like diet. No purposive or selective sampling was performed; rather, the study included all eligible cases available in the electronic record system within the defined study period. Due to the retrospective nature of the study, some variables were incompletely documented in the medical records. Therefore, analyses were performed using the available data for each outcome, and the effective sample size varied across tables and figures depending on data completeness for adherence, seizure duration, seizure control, adverse effects, and follow-up measures.

### 2.4. Variables

Data were obtained from the TrakCare electronic medical record system at Sultan Qaboos University Hospital (SQUH). This system provided access to patient demographics, clinical characteristics, medication records, and follow-up data. Data extraction was performed using standardized data collection forms developed for this study. Medication adherence was assessed using the Medication Possession Ratio (MPR), calculated from pharmacy refill records. Statistical analysis was conducted using IBM SPSS Statistics software (version 26) and Microsoft Excel for data management and visualization. No laboratory equipment or experimental materials were used, as this was a retrospective study based on existing clinical records.

### 2.5. Adherence Assessment

Data were extracted retrospectively from the TrakCare electronic medical record system. Information collected included demographics (age and sex), clinical history (onset of seizure, duration of the attack, type of epilepsy, seizure frequency and severity, and seizure-free duration), family history, genetic disorders, and comorbidities. Details on ASMS therapy included the drug name(s) and any modifications, such as dose changes, discontinuation, or switching of medications during follow-up. Adherence was obtained from pharmacy refill records and was assessed based on the Medication Possession Ratio (MPR), which is a validated metric in pharmacoepidemiologic research. MPR is calculated using the formula:

$$\mathrm{MPR}=\left(\frac{\mathrm{Sum}\;\mathrm{of}\;\mathrm{days}’\;\mathrm{supply}\;\mathrm{for}\;\mathrm{all}\;\mathrm{fills}\;\mathrm{in}\;\mathrm{period}}{\mathrm{Number}\;\mathrm{of}\;\mathrm{days}\;\mathrm{in}\;\mathrm{period}}\right)\times 100\%$$

An MPR value of ≥80% was considered indicative of good adherence [5].

Side effects were recorded as documented in clinical notes or caregiver complaints, including behavioral, cognitive, or physical symptoms. Additional variables included sleep pattern (reported as usual, increased, or disturbed) and treatment outcome, with seizure control based on documented seizure-free periods of at least one year [13].

### 2.6. Ethical Considerations

This study was approved by the Medical Research Ethics Committee (MREC) at the College of Medicine and Health Sciences, Sultan Qaboos University Hospital (SQUH) under ethical approval SQU-EC/232\2024-MREC #3416. All data were anonymyzed before analysis to ensure confidentiality and privacy. Under institutional guidelines for studies using de-identified retrospective data, informed consent was not required.

### 2.7. Bias and Confounding Control

Several measures were implemented to minimize potential bias in this study. Selection bias was reduced by applying predefined inclusion and exclusion criteria and by including all eligible patients within the study period. Information bias was minimized through the use of standardized electronic medical records (TrakCare), ensuring consistent and reliable data collection. To reduce measurement bias, adherence was assessed using an objective and validated metric (Medication Possession Ratio), and seizure outcomes were based on documented clinical records. Outcome assessment bias was minimized using predefined criteria for seizure control and seizure-free duration. Potential confounding factors, including age, sex, and type of epilepsy, were accounted for during data analysis using stratification and comparative statistical methods. Additionally, statistical analyses were performed using appropriate tests (e.g., chi-square test), and results were interpreted based on predefined significance levels (*p* ≤ 0.05).

### 2.8. Statistical Analysis

All data collected were entered and analyzed using SPSS software (version 26) and Microsoft Excel. Descriptive statistics, including mean, range, standard deviation (SD), frequency, and percentages, were used to summarize patient demographics, ASMS usage, seizure control, adherence patterns, and adverse effects. Cross-tabulations were conducted for categorical variables, such as adherence status, duration of attacks, seizure control, and side effects. The chi-square test assessed statistical significance between categorical groups, such as comparing seizure control rates between patients with and without side effects. Results were considered statistically significant at a *p*-value ≤ 0.05. Visual tools such as bar charts, pie charts, and frequency tables were used to present the results clearly and support interpretation.

## 3. Results

### 3.1. ASMS Utilization

**Figure 1:**
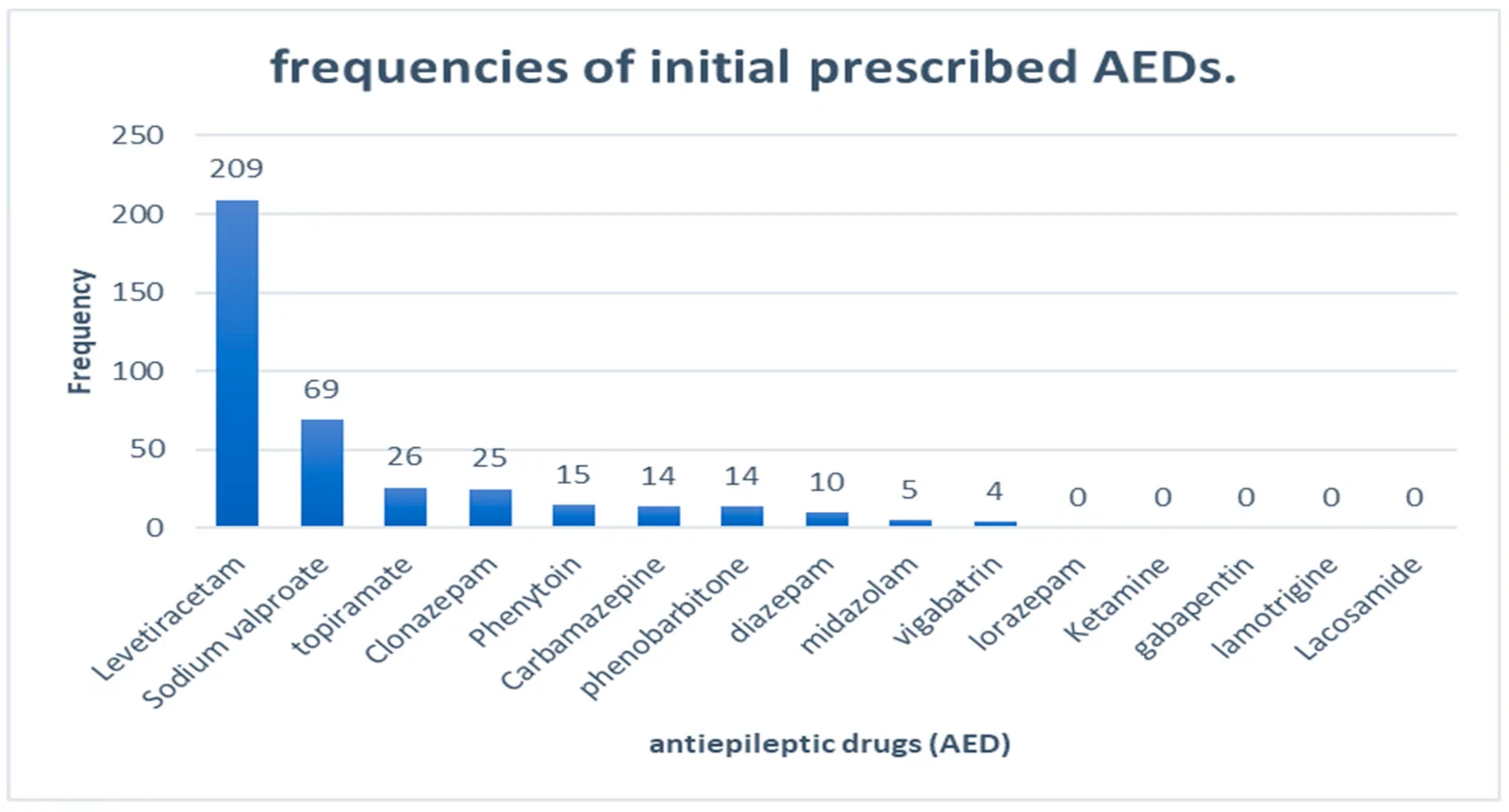
Frequency of initial prescribed anti-seizure medications (ASMs) at diagnosis.

The full cohort included 305 patients; however, the analytic denominator varied across results according to the availability of outcome-specific data. The sample size used for each analysis is specified in the corresponding table or figure. Upon diagnosis, levetiracetam was the most frequently recommended medicine, constituting 68.52% of prescriptions. The prescription of sodium valproate (22.62%) was subsequently followed by topiramate (8.52%) (Figure 1).

This figure shows the distribution of ASMs prescribed at the time of diagnosis among pediatric patients with epilepsy. Levetiracetam was the most frequently prescribed medication, followed by sodium valproate and Topiramate.

### 3.2. Anti-Seizure Medication Use and Adherence During Follow-Up

**Figure 2:**
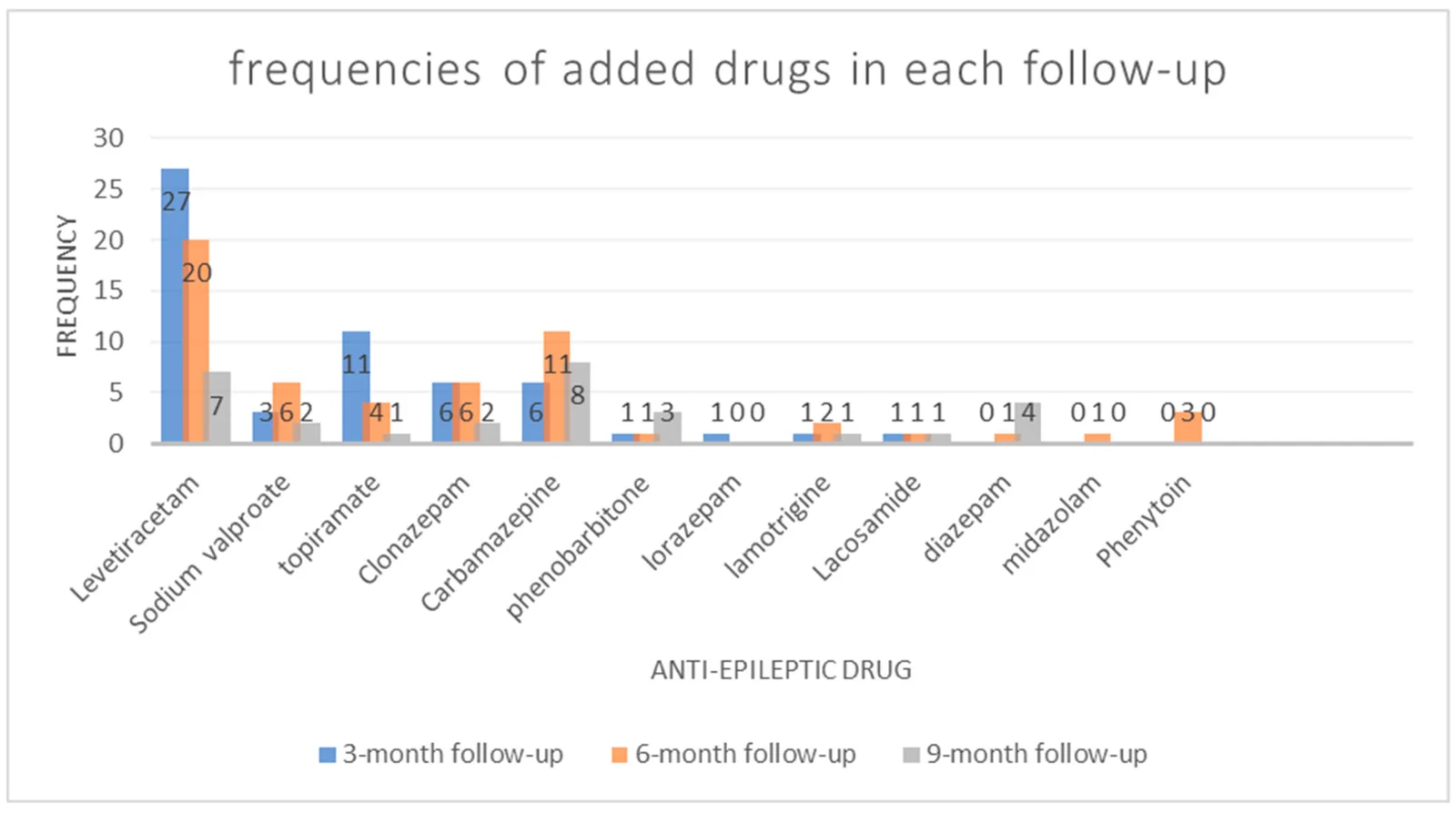
Distribution of additional anti-seizure medications across follow-up visits.

In the 3-month follow-up, the medications most frequently added were levetiracetam (8.85%), topiramate (3.61%), and clonazepam (1.97%). At the 6-month follow-up, levetiracetam continued to be the most added drug (6.56%), followed by carbamazepine (3.61%), sodium valproate (1.97%), and clonazepam (1.97%). On the other hand, in the 12-month follow-up, carbamazepine was the most frequently added medication (2.62%), followed by levetiracetam (2.30%) and diazepam (1.31%) (Figure 2).

This figure illustrates the frequency at which ASMs were added during follow-up at 3, 6, and 12 months. Levetiracetam was the most commonly added medication across all follow-up intervals, with variations observed for other ASMs.

After excluding the missing data (27 cases) due to the fact that refill prescriptions are administered from local hospitals, approximately half of the patients were classified as adherent, while the remainder were non-adherent (Table 1).

This table presents the number and percentage of pediatric patients classified as adherent (MPR ≥ 80%) and non-adherent (MPR < 80%) to anti-seizure medications. After excluding 27 patients with unavailable refill data, 54.68% of patients were classified as adherent and 45.32% were classified as non-adherent based on the Medication Possession Ratio.

### 3.3. The Factors Influencing the Selection of ASMSs, Including Patient Demographics and Epilepsy Types

Patients aged 6–12 demonstrated an adherence rate of 37.41% to anti-seizure medications (ASMSs), compared to 17.26% in the younger age group (*p* = 0.123). This difference is not statistically significant. Additionally, there is no significant relationship between sex and level of adherence (*p* = 0.668). There is no significant difference between anti-seizure and rescue medications recorded at diagnosis with adherence status, as shown in Table 2.

This table shows the relationship between age, sex, and type of initially prescribed ASM with adherence status. Data are presented as frequency (percentage), and *p*-values were calculated using the chi-square test.

The comparison between the valproate and levetiracetam groups showed no significant difference in mean age, with the valproate group having a mean age of 7.41 ± 2.85 years and the levetiracetam group having a mean age of 6.89 ± 2.82 years (*p* = 0.296). Regarding gender, 52.2% of patients in the valproate group were female, compared to 45.5% in the levetiracetam group (*p* = 0.332). A significant difference was found in seizure types (*p* = 0.041), with 59.4% of patients in the valproate group experiencing generalized seizures, compared to 46.4% in the levetiracetam group. Conversely, 53.6% of patients in the levetiracetam group had focal seizures, compared to 40.6% in the valproate group (Table 3).

Levetiracetam, sodium valproate, and topiramate were the most commonly prescribed maintenance ASM. Diazepam was used as a rescue medication in acute seizure management and was therefore considered separately from maintenance therapy.

This table compares age, sex distribution, and seizure type between patients receiving valproate and those receiving levetiracetam. Continuous variables are presented as the mean ± standard deviation, and categorical variables are presented as the frequency (percentage).

### 3.4. Seizure-Free Duration and Seizure Severity in Pediatrics Receiving ASMSs

**Figure 3:**
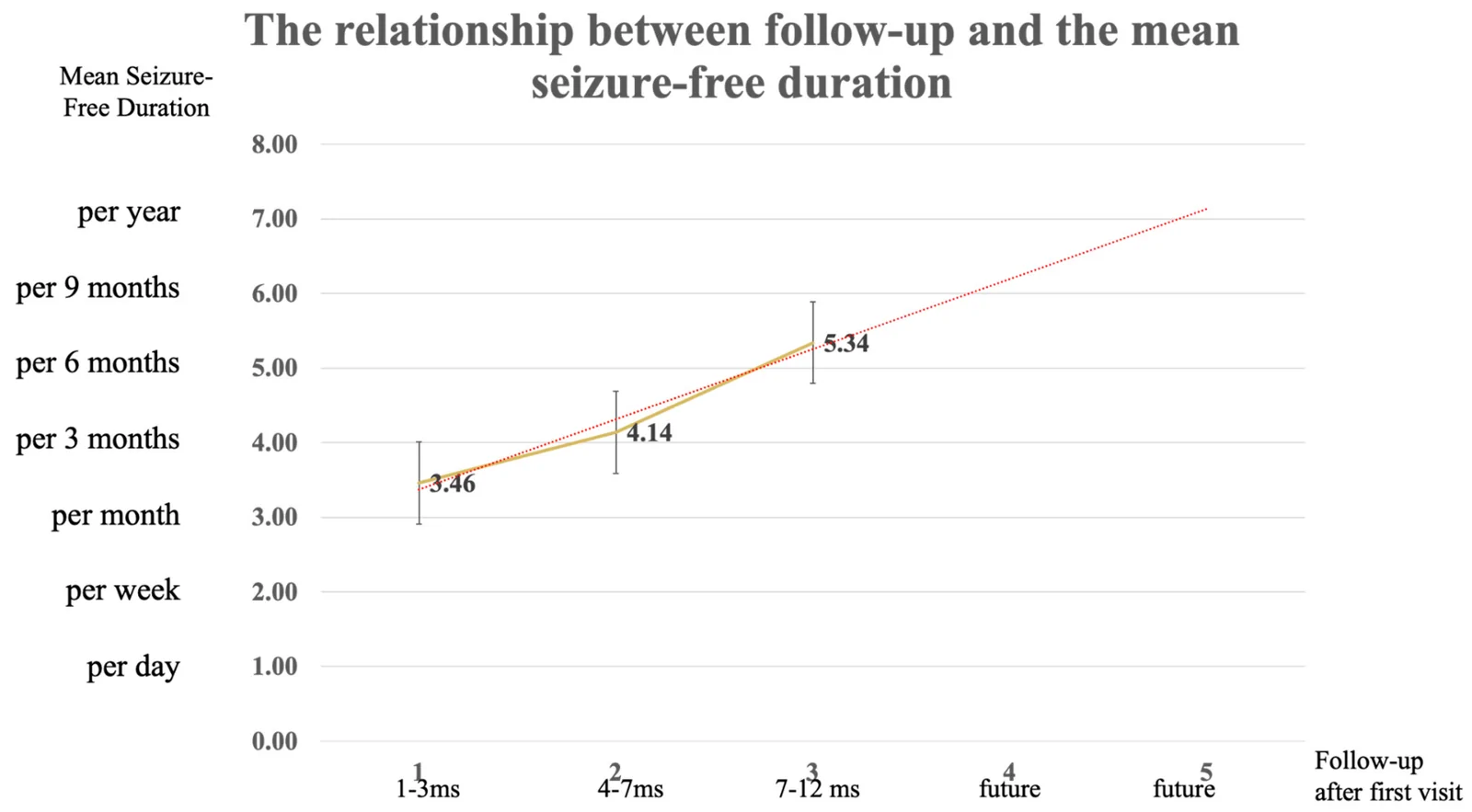
Change in mean seizure-free duration across follow-up visits in pediatric patients receiving ASMs.

The mean seizure-free duration at the first visit is 3.46, at the second visit is 4.14, and at the third visit is 5.34. The mean seizure-free duration increased progressively with each follow-up. The slight upward slope in the graph suggests that ASMS therapy led to a reduction in seizure occurrences over time, with improved patient outcomes over the 2-year follow-up period. The red dotted line indicates the expected reduction in seizure duration in future follow-ups (Figure 3).

This figure demonstrates the increase in mean seizure-free duration over successive follow-up visits. The *y*-axis represents the mean seizure-free duration in months. Based on patients with complete seizure-duration data across the relevant follow-up visits (n = 216), mean seizure-free duration increased progressively, from 3.46 months at the first visit to 4.14 months at the second visit and 5.34 months at the third visit.

The mean seizure duration showed a clear reduction over the 2-year follow-up period among 216 patients, out of a total of 305. Eighty-nine patients were excluded due to missing seizure-related information in their medical records for at least one of the three follow-up visits. At the first visit, the mean seizure duration was 3.43 min. This decreased to 2.24 min at the first follow-up (1–3 months), then to 1.67 min at the second follow-up (4–7 months), and 1.26 min at the third follow-up (7–12 months). This progressive reduction in seizure duration highlights the effectiveness of continued ASMS therapy in managing seizure severity over time (Table 4).

This table shows the change in mean seizure duration (minutes) at baseline and during follow-up visits (7–12 months). Analysis included 216 patients with complete seizure-duration records across baseline and follow-up visits.

At the first visit, 86.3% (n = 214) of patients experienced seizures lasting more than one minute, while only 13.7% (n = 34) had seizures lasting less than one minute. By the third follow-up, the proportion of patients with seizures lasting more than one minute had decreased to 60.1% (n = 149), whereas those with shorter seizures increased to 39.9% (n = 99), as shown in Table 5. This notable shift suggests a reduction in the frequency of prolonged seizures, reflecting the potential effectiveness of ASMS therapy over the two-year follow-up period. However, the *p*-value of 0.180 indicates that this change in seizure duration is not statistically significant, which means the observed difference could be due to random variation. The analysis included data from 248 patients; 57 patients were excluded due to incomplete seizure-related information in their medical records (Table 5).

This table compares the proportion of patients with seizure duration greater than one minute versus less than or equal to one minute at baseline and at the final follow-up. Statistical significance was assessed using the chi-square test. Analysis included 248 patients with complete baseline and final follow-up seizure-duration category data; 57 patients were excluded because of incomplete seizure documentation.

Levetiracetam showed a statistically significant reduction in the duration of an attack, with a *p*-value of <0.001, reflecting a significant transition from prolonged to shorter seizure durations over time. The value for sodium valproate was *p* = 0.040. On the other hand, topiramate (*p* = 0.80) showed no statistically significant differences. Overall, the results suggest that levetiracetam, sodium valproate, and phenytoin may be more effective in reducing seizure duration in pediatric patients compared to other ASMSs (Table 6).

This table presents the distribution of seizure duration categories (>1 min vs. ≤1 min) for selected ASMs at baseline and final follow-up, along with corresponding *p*-values. Levetiracetam showed a statistically significant reduction in seizure duration over time.

### 3.5. The Prevalence of Adverse Effects Associated with ASMS Use and Their Impact on Treatment Adherence and Seizure Control

In a group of 305 patients using an epilepsy drug, the most common side effects were fatigue (29.8%), dizziness (24.3%), headache (19.3%), and lethargy (17%). Cognitive or behavioral issues like delayed speech (11.8%) and hyperactivity (9.2%) were also reported. Rare but serious effects, such as depression and suicidal thoughts, occurred in only 0.7%. (Table 7).

Patients could experience and report more than one adverse effect; therefore, the total frequency exceeds the number of patients included in the study (n = 305). Because some patients experienced more than one adverse effect, the categories shown in Table 7 are not mutually exclusive, and the total frequency exceeds the study sample size.

### 3.6. The Proportion of Patients Achieving Seizure Control with Their Current ASMS Regimen

The data shows that only 43% of patients achieved seizure control, while 57% did not, highlighting a significant gap in treatment effectiveness and the need for improved management strategies. (Table 8).

This table presents the number and percentage of patients classified as having controlled versus uncontrolled seizures.

Seizure control was attained in 131 out of 305 patients, or 43%. Of those who did not encounter side effects, 56.2% attained seizure control. Conversely, merely 10.2% of individuals who had side symptoms were regulated. The difference was statistically significant (*p* < 0.001, Chi-square test), indicating a strong association between the absence of adverse effects and better seizure control outcomes (Table 9).

This table shows the relationship between the presence of ASM-related adverse effects and seizure control status. Data are presented as the frequency (percentage), and statistical significance was assessed using the chi-square test.

Among the study sample of 305 patients, a predominant number of those who had side effects (n = 88) were inadequately controlled, with 89.8% (n = 79) classified as “Not Controlled” and merely 10.2% (n = 9) attaining seizure control. Conversely, among patients without side effects (n = 217), a greater percentage attained seizure control—56.2% (n = 122) were controlled, whereas 43.8% (n = 95) were not. The results indicate a robust correlation between a lack of side effects and improved seizure control outcomes.

Among 278 patients, 82 experienced side effects, while 196 did not. A greater proportion of patients without side effects (57.7%) adhered to their treatment, compared to 42.3% who did not. In contrast, among those with side effects, adherence dropped to 47.6%, while non-adherence increased to 52.4%. These findings suggest that while the presence of side effects may reduce medication adherence in individuals with epilepsy, the impact is less pronounced than the effect observed on seizure control outcomes. (Table 10).

This table presents the relationship between the presence of adverse effects and adherence status among pediatric patients receiving ASMs. Analysis included 278 patients; 27 were excluded because adherence could not be calculated from refill records.

This table displays the relationship between reported side effects of anti-seizure drugs (ASMSs) and treatment adherence.

### 3.7. Improve Epilepsy Management by Optimizing ASMS Dosage Adjustments Across Follow-Up Visits

To identify opportunities to improve epilepsy management by optimizing ASMS dosage adjustments across follow-up visits, Table 11 highlights the impact of dose changes on the occurrence of side effects in both controlled and uncontrolled epilepsy groups. In the uncontrolled epilepsy group, increasing the dose led to 53.8% of patients experiencing side effects, while decreasing the dose reduced the side effects to 46.7%. In the controlled epilepsy group, 69.1% experienced side effects after an increase in dose, and 44.4% experienced side effects after a decrease in dose. The *p*-value of 0.044 indicates a statistically significant relationship between dose adjustments and the occurrence of side effects, suggesting that dose changes significantly influence side effect outcomes in epilepsy treatment.

This table illustrates the relationship between dose changes (increase or decrease) and the occurrence of adverse effects in patients with controlled and uncontrolled epilepsy. Statistical significance was assessed using the chi-square test.

## 4. Discussion

### 4.1. Most Prescribed ASMs

Currently, there is a paucity of data regarding the utilization trends of anti-seizure medications (ASMs) in juvenile populations in Oman. This study identifies levetiracetam as the most commonly prescribed antiepileptic medication, followed by sodium valproate and topiramate. This discovery indicates a transition to newer-generation ASMs characterized by enhanced safety and tolerability profiles. Previous research has indicated varying prescription patterns. Research conducted in Jordan and Europe indicates an increased usage of sodium valproate [14,15]. The growing preference for levetiracetam in our study can be attributed to its advantageous pharmacokinetic characteristics, reduced likelihood of drug–drug interactions, and superior tolerability, especially in young patients [4].

### 4.2. ASMS Effectiveness

In this study, adherence to antiepileptic medications was modest, with slightly more than half of the patients deemed adherent. No substantial correlation was detected between adherence and seizure management. Although adherence is typically regarded as a crucial aspect in treatment success, this data indicates that other elements may affect outcomes in pediatric epilepsy.

Previous research has indicated varied adherence rates and influences on seizure management. For example, elevated adherence rates were observed in Germany [16], and other studies have demonstrated a positive correlation between adherence and enhanced clinical outcomes [17,18]. The absence of correlation seen in this study may stem from utilizing prescription refill data as a surrogate for adherence, which does not accurately reflect real medicine usage. Moreover, elements such as drug acceptability and seizure classification may exert a more significant influence on outcomes.

### 4.3. Selection of ASMSs Based on Patient Demographics and Epilepsy Types

This study revealed that the selection of ASM was not substantially affected by demographic factors, including age and sex. Nonetheless, the type of seizure was strongly correlated with the selection of anti-seizure medication. Patients with widespread seizures were more often administered sodium valproate, while those with focal seizures were more commonly provided with levetiracetam. These findings align with contemporary clinical guidelines and previous research, which underscore the importance of customizing ASM selection according to seizure type and clinical attributes [19]. This indicates that the prescribing practices in the study environment conform to established therapeutic guidelines.

### 4.4. Seizure-Free Duration and Seizure Severity

The findings of this study indicate a gradual decrease in seizure duration over time, alongside an improvement in seizure-free periods during follow-up visits. The data indicate that ongoing ASM therapy enhances seizure management in pediatric patients. Previous research has indicated analogous findings, demonstrating that continued care and prompt intervention correlate with improved long-term outcomes [20]. The observed transition from prolonged to abbreviated seizure durations may indicate successful optimization of treatment protocols during follow-up. The early and consistent administration of antiepileptic medications results in a reduction in seizure severity and an improvement in overall prognosis [7].

### 4.5. Seizure Control and Side Effects

This study reveals a significant correlation between a lack of adverse effects and enhanced seizure management. Patients devoid of reported side effects were markedly more inclined to attain seizure control than those experiencing negative signs and symptoms. This discovery aligns with the current literature, which emphasizes the influence of side effects on treatment outcomes. Adverse effects may necessitate a dose decrease, diminish adherence, or result in therapy termination, consequently jeopardizing seizure control [21]. These findings underscore the need to choose ASMs with advantageous tolerability characteristics.

### 4.6. Adherence and Side Effects

Despite the association of unfavorable effects with marginally reduced adherence rates, the link lacked statistical significance. This indicates that although tolerability may affect adherence, its impact may differ based on the individual patient and caregiver characteristics. Previous research has shown that unpleasant effects strongly contribute to non-adherence and suboptimal outcomes [22]. Conversely, the results of this study may indicate the influence of caregiver oversight on juvenile populations, where medication compliance may persist despite minor or controllable side effects.

### 4.7. Optimizing ASMS Dosage Adjustments Across Follow-Up Visits

This study found a substantial correlation between dose modifications and the incidence of adverse events. Elevating ASM dosages correlated with an increased incidence of side effects, while a decrease in dosage was connected with a diminished occurrence of adverse events. These findings underscore the necessity of meticulous dose titration to reconcile therapy efficacy with tolerability. While dose escalation is frequently required for effective seizure management, excessive increments may result in side effects that undermine overall therapeutic efficacy. This highlights the necessity for personalized treatment methods, especially in young patients, where physiological variability and developmental variables can affect drug response.

The correlation between dose modifications and treatment results may potentially be affected by pharmacological resistance. A subset of patients with epilepsy developed pharmacoresistance, which restricts the efficacy of antiepileptic drug therapy [21].

From a therapeutic standpoint, these data underscore that improving ASM therapy necessitates not only attaining sufficient seizure control but also mitigating side effects. Meticulous oversight and personalized dosage modifications are crucial for enhancing therapeutic results and patient well-being. These findings underscore the significance of personalized dose titration in the therapy of epilepsy. Previous studies have provided analogous data, indicating that dose escalation correlates with heightened deleterious effects [4,23,24,25]. Meticulous dose optimization is crucial to achieving a balance between efficacy and tolerability, especially in young patients. The observed reduction in seizure duration over follow-up may suggest improved seizure outcomes during treatment, but causal inferences should be made cautiously because of the retrospective observational design and limitations in longitudinal statistical modeling.

Previous research has shown that unpleasant effects significantly contribute to non-adherence and suboptimal outcomes [22]. Conversely, the results of this study may indicate the influence of caregiver oversight on juvenile populations, where medication compliance may persist despite minor or manageable adverse effects. Diazepam should be interpreted as a rescue medication used for acute seizure control rather than as a routine maintenance ASM. Ketamine is not classified as a standard ASM in chronic epilepsy management.

Our findings should also be interpreted in the context of the broader literature on pharmacoresistant epilepsy, which emphasizes that treatment response is shaped by multiple interrelated factors, including epilepsy syndrome, seizure type, underlying etiology, prior treatment failure, and overall treatment burden. This may partly explain the heterogeneity of outcomes observed in the present study and highlight the importance of cautious interpretation of treatment-related associations in routine clinical practice.

## 5. Limitations of the Study

- Patients were lost to follow-up because they sought medical advice outside Oman.
- Prescription refills do not reflect the actual consumption of medications during a specific period, and not all prescriptions are collected from the SQUH pharmacy.
- Side effect reporting, primarily based on caregiver and clinician observations, may have been affected by recall bias.
- The findings may not be generalizable to adolescents with epilepsy, as patients aged 13–17 years were not included in the present study.
- The one-year seizure-free threshold used in this study was an operational outcome definition based on available follow-up data and may differ from guideline-based seizure-freedom criteria applied to decisions about anti-seizure medication withdrawal.
- Given the retrospective observational design and absence of a comparison group, the findings should be interpreted as associations rather than evidence of causal treatment effects.
- Future prospective multicenter studies with standardized outcome assessment and broader pediatric age representation are needed to confirm these findings.
- The observed association between adverse effects and seizure control should be interpreted cautiously, as it may reflect confounding by indication. Patients with more severe or treatment-resistant epilepsy are often exposed to greater treatment intensity, which may increase both the risk of adverse effects and the likelihood of poorer seizure outcomes. Because factors such as drug load and epilepsy severity were not adjusted for, this finding cannot be interpreted as an independent association.

## 6. Future Research

To improve epilepsy management by optimizing ASMS dosage adjustments, future prospective research should be conducted to enable real-time tracking of changes in dosing, side effects, and seizure control. Incorporating quality-of-life measures and serum drug level monitoring may offer a more holistic evaluation of treatment effectiveness. Additionally, examining the influence of early treatment initiation and adherence patterns on seizure control, along with relevant social factors like parental education and household income, may provide a deeper understanding of treatment outcomes. Finally, integrating pharmacogenomic data into future studies could help predict which patients are more likely to benefit from certain ASMSs or experience adverse effects, supporting more personalized and effective epilepsy management. Published Oman-based data on anti-seizure medication utilization are available for some populations, including adults; however, pediatric-specific evidence remains limited. In this context, the present study helps address an important local knowledge gap regarding prescribing patterns, adherence, adverse effects, and seizure outcomes in children with epilepsy.

## 7. Conclusions

In conclusion, levetiracetam is the most widely used drug in SQUH. Demographics and type of ASMS have no significant influence on adherence level. Factors influencing ASMS selection were found to be primarily seizure type, with valproate prescribed for generalized seizures and levetiracetam prescribed for focal seizures, consistent with clinical guidelines. Also, ASMSs had a role in prolonging seizure-free duration and reducing seizure severity. The findings indicate that seizure control remains suboptimal in many pediatric patients, suggesting the need for more effective management approaches. Adverse effects were found to negatively affect both treatment adherence and seizure control, underscoring the importance of improving drug tolerability in epilepsy management. Finally, dose adjustments over time showed that increasing doses often led to more side effects and lower seizure-free rates. While the present study reflects practice within Oman, its findings may have broader relevance because many of the identified issues, including variation in anti-seizure medication use, treatment-related challenges, and the need for individualized therapeutic strategies, are encountered internationally. Nevertheless, differences in healthcare infrastructure, medicine availability, and local prescribing practices should be considered when interpreting the generalizability of these results.

## Figures and Tables

**Table 1:** Distribution of adherence based on Medication Possession Ratio (MPR).

State of Adherence	Frequency
Adherent	(154) 54.68%
Non-adherent	(126) 45.32%

**Table 2:** Association between patient demographics, initial anti-seizure medications, and adherence status.

State of Adherence	Non-Adherent	Adherent	*p*-Value
**Age (yr.)**			**0.123**
e ≤ 5	51 (18.34%)	48 (17.26%)	
e > 6	75 (26.13%)	104 (37.41%)	
**Sex**			**0.668**
Female	56 (20.12%)	71 (25.53%)	
Male	70 (25.17%)	80 (28.77%)	
**Anti-seizure and rescue medications recorded at diagnosis**			
Diazepam	6 (2.15%)	3 (1.07%)	0.191
Midazolam	3 (1.07%)	1 (0.35%)	0.230
Levetiracetam	89 (32.01%)	106 (38.12%)	0.871
Phenobarbitone	7 (2.51%)	3 (1.07%)	0.110
Sodium valproate	26 (9.35%)	37 (13.30%)	0.462
Clonazepam	15 (5.39%)	7 (2.51%)	0.025
Topiramate	8 (2.87%)	17 (6.11%)	0.161
Carbamazepine	5 (1.79%)	8 (2.87%)	0.611
Phenytoin	6 (2.15%)	7 (2.51%)	0.951
Vigabatrin	2 (0.71%)	1 (0.35%)	0.455

**Table 3:** Comparison of demographic and clinical characteristics between valproate and levetiracetam groups.

Parameter	Valproate Group (n = 69)	Levetiracetam Group (n = 209)	*p*-Value
**Mean age (yrs.)**	7.41 ± 2.853	6.89 ± 2.822	0.296
**Gender**			0.332
- Female	36 (52.2%)	95 (45.5%)	
- Male	33 (47.8%)	114 (54.5%)	
**Types of seizure**			0.041
- Generalized seizures	41 (59.4%)	97 (46.4%)	
- Focal seizures	28 (40.6%)	112 (53.6%)	

**Table 4:** Mean seizure duration across baseline and follow-up visits.

Times (Months)	Mean Duration of Attack (min)
First Visit	3.43
First Follow-up 1–3	2.24
Second Follow-up 4–7	1.67
Third Follow-up 7–12	1.26

**Table 5:** Comparison of seizure duration categories at baseline and final follow-up.

	First Visit		Third Follow-Up		*p*-Value
	(n)	%	(n)	%	
**More than 1 min**	214	86.3%	149	60.1%	**0.180**
**Less than 1 min**	34	13.7%	99	39.9%	
**Total**	248	100%	248	100%	

**Table 6:** Association between anti-seizure medication type and seizure duration over time.

ASMS	Time	Duration of the Attack		Total (n) Prescribed Drugs
		>1 min (n)	≤1 min (n)	
**Levetiracetam**	**First visit**	155	16	171
	**Third follow-up**	91	80	
**Sodium valproate**	**First visit**	46	12	58
	**Third follow-up**	34	24	
**Topiramate**	**First visit**	21	4	25
	**Third follow-up**	20	5	
				254

**Table 7:** Frequency and percentage of reported adverse effects among pediatric patients receiving ASMs.

Adverse Effect	Frequency (n) (Total = 305)	Percent %
depression	2	0.7
lethargy	52	17
ataxia	20	6.6
dizziness	74	24.3
appetite changes	35	11.5
numbness	19	6.2
fatigue	91	29.8
bone pain	8	2.6
aggressive	10	3.3
headache	59	19.3
sedation	33	10.8
GI disturbances	35	11.5
weight gain	4	1.3
hyperactivity	28	9.2
difficulty in learning or delayed speech	36	11.8
nystagmus	13	4.3
biting nails	22	7.2
rash	19	6.2
suicidal thoughts	2	0.7
nausea	31	10.2

**Table 8:** Proportion of patients achieving seizure control.

Seizure Control	Frequency (%)
**Controlled**	131 (43.0%)
**Not controlled**	174 (57.0%)

**Table 9:** Association between adverse effects and seizure control.

Side Effects and Seizure Control					
			Not Controlled	Controlled	Total
**Side Effects (SEs)**	**Without SEs**	**n**	95	122	217
		**%**	43.8%	56.2%	100.0%
	**With SEs**	**n**	79	9	88
		**%**	89.8%	10.2%	100.0%
**Total**		**n**	174	131	305
		**%**	57.0%	43.0%	100.0%
***p*** **-value**				<0.001	

**Table 10:** Association between adverse effects and medication adherence.

Side Effects and Adherence Status					
			Not Adherent	Adherent	Total
	**Without SEs**	**n**	83	113	196
		**%**	42.3%	57.7%	100.0%
	**With SEs**	**n**	43	39	82
		**%**	52.4%	47.6%	100.0%
**Total**		**n**	126	152	278
		**%**	45.3%	54.7%	100.0%
***p*** **-value**				0.159	

**Table 11:** Impact of anti-seizure medication dose adjustments on adverse effects.

Group	Side Effects		
	NO	YES	Total Count
**Uncontrolled epilepsy group (High dose)**	48 (46.2%)	56 (53.8%)	104
**Controlled epilepsy group (High dose)**	34 (30.9%)	76 (69.1%)	110
**Uncontrolled epilepsy group (Low dose)**	32 (53.3%)	28 (46.7%)	60
**Controlled epilepsy group (Low dose)**	20 (55.6%)	16 (44.4%)	36

## Data Availability

The data presented in the study are available on request from the corresponding author. Of the 305 included patients, adherence analysis was available for 278 patients because 27 obtained refills outside the study’s pharmacy system. Seizure-duration category comparisons included 248 patients with complete first-visit and final follow-up data, while repeated seizure-duration analysis across all visits included 216 patients with complete documentation at all required time points. Analyses of seizure control and side effects were based on records with complete outcome documentation.
